# Round the Hospitals

**Published:** 1920-09-11

**Authors:** 


### ROUND THE HOSPITALS.

Great relief is experienced by the nursing staff of the London Hospital at the more favourable turn in the finances of the institution. While no one seriously believed in the closing down of the London altogether, it did seem not long ago that the drastic step of closing a wing containing 300 beds might be forced upon the Committee. The amount of dislocation created by such a disaster can be readily understood by our readers. Quite one hundred nurses would have been affected; much suffering would have been entailed; the already long waiting list would have swelled beyond bounds, and a general feeling of insecurity would have ensued. The introduction of payment for in-patients at a rate of a guinea a week, with free treatment for children under seven, has been accomplished with wonderfully little difficulty. Many patients are actually glad to pay. Few are found incapable of doing so. No urgent

**ROUND THE HOSPITALS—*(continued)*.**

cases are refused, and relatives and friends readily find means to defray the cost when appealed to. We believe that hospital managers will find their voluntary subscribers more than acquiescent in the system of payment by patients. It is the consciousness that the majority of patients are fully able to contribute to the cost of their treatment which has caused something like a revolt among hospital subscribers with limited incomes.

The groundwork of British and American kinship and friendship is being brought before the dwellers on both sides of the Atlantic in the most picturesque manner possible this week by the Mayflower celebrations at Plymouth. Three centuries ago, when the Pilgrim Fathers left our shores, philanthropy towards the under dog, the criminal, the lunatic, the defective, the fever-stricken, the chronically sick, and incurable had not yet stirred more than isolated individuals here and there. It saw its rise in America and in this country much about the same time. It is now one of the firmest links which unites the two nations. A noble emulation in the relief of suffering has done more to draw the English-speaking races together than can be undone by the politicians. During the war in many parts of the world, English and American nursing sisters worked together and learned to trust each other as only women can who are living up to the top of their powers in extremity of danger. The fruitful co-operation of America and Great Britain in helping to abolish the manifold evils left behind by the war is happily secured through the International Red Cross organisation of the League of Nations, and cannot be disturbed.

We are officially informed that, with a view to facilitating the more rapid distribution of war decorations, no more recipients of the Royal Red Cross (Second Class), or of certain other decorations awarded in connection with the war, will be summoned to an Investiture held by the King personally. All those to whom this decoration has been awarded will have the option of receiving it at an investiture in the county in which each may reside or of having it sent by post. The county investiture referred to will be held by the Lord Lieutenant, but only if it is found that a sufficient number of recipients desire to be present at the ceremony. The first step to be taken is for each person concerned to write to the Secretary, C. 2, Investitures, War Office, Whitehall, giving her full name and postal address (specifying the county) and particulars of her service by which she can be identified, and stating whether, in the circumstances, she wishes to receive the insignia by post or at a county investiture.

Nursing members of voluntary detachments will be glad of the rise in their pay recently announced in an Army Council instruction. All serving on August 17 are to be entitled to three shillings a week extra as from May 1. The increase is meant to aid members under the high cost of living, and does not apply to those accommodated in billets or hostels under War Office arrangements or the "approved lodging" scheme.

Mr. Arthur Balfour has, in the name of the Council of the League of Nations, appealed to the Governments linked with it for an immediate grant of £250,000 out of the £10,000,000 needed to stem the typhus epidemic in Poland and Galicia. Frequent warnings have been issued from the League of the grave dangers which menace Europe from this plague spot in the coming winter. Medical authorities who have visited Russia declare that typhus has swept that country from end to end, that scarcely a town or village has escaped, and that half the doctors who have been on duty in these areas have succumbed to it. The disease is being carried further by returning prisoners and by refugees. To combat it strong and efficient measures need to be taken at once. The Red Cross Societies of Europe and America, linked together under the organisation of the League of Nations, have prepared a plan of action and have large resources. With an immediate grant from the Governments, medical necessities can be obtained, with clothing and suitable provision of other kinds for the sick. But there is no time to lose.

The New End Hospital at Hampstead has just started on its new career as a finely-equipped municipal hospital after five years' occupation by the military authorities for the treatment of the wounded. In addition to its uses as an infirmary for the sick and for chronic invalids, it is to serve the purpose of a hospital to which other boroughs may send cases needing special treatment, and residents of the borough will also be received as paying patients. The resident medical officer is Dr. Reade. The nursing department is under Miss M. Jackson, O.B.E., R.R.C., who was appointed matron some months ago. The nurses' quarters are particularly comfortable, charmingly decorated, and well furnished. During the war the inmates were accommodated at the Marylebone Infirmary, and they have been very impatient to return to their old quarters under the altered conditions. They are of all ages, from new-born infants to old folk well on in their eighties, but all got through their journey well and have settled down contentedly. Mrs. Munro, the present Chairman of the Board of Guardians, visited the hospital and helped to receive them.

This is the time of year when hospital fêtes abound and bring much consolation to the hearts of anxious treasurers. One of the most striking recently held was that in the grounds of Walmer Castle, by kind permission of Lord and Lady Beauchamp. Princess Mary Louise opened the bazaar, and a large and distinguished company assembled. The object is to build a new hospital for Walmer and Deal as a war memorial, for which purpose £12,000 has already been subscribed, and the successful organisation of the fête has, we understand, resulted in securing a large proportion of the £2,000 still required.

**ROUND THE HOSPITALS**—(*continued*).

Crumpsall Infirmary, Manchester, which has always been in the van of progress, has again strengthened its position as one of the foremost nurse-training schools in the Poor-Law service at its best. It has appointed to the post of sister-tutor Miss Norah McCheane, the winner in 1919 of the Cowdray Scholarship. Miss McCheane was trained at the Royal Sussex County Hospital, Brighton, and subsequently held the post of ward-sister at Seaford and at the Dorset County Hospital, and sister-in-charge of the *x*-ray and electrical department at Addenbrooke's Hospital, Cambridge. She holds the honours certificate of the Incorporated Society of Trained Masseuses, and during the past year has been taking the sister-tutor's course at King's College for Women. She has already begun her duties at Crumpsall. We congratulate the Guardians, the matron, Miss Burgess, and the Crumpsall probationers on this appointment, which is one of great interest. West Ham was, we believe, the first Poor-Law infirmary to appoint a sister-tutor to its staff, and we hope that in the near future this officer will be considered an indispensable member of the staff of every recognised nurse-training school.

The Order of St. John of Jerusalem have inaugurated an entirely new movement in presenting the history of Florence Nightingale in series of moving pictures, and using it for the purpose of attracting suitable women to become nurses. We have more than once advocated this policy in the present scarcity of good candidates. Ambulance Officer C. Hanmer, official lecture demonstrator to the Ambulance Department of St. John, is responsible for the scenario, in which Miss Elizabeth Risdon assumed the leading part. The film was shown recently at Lincoln by Mr. Hanmer, and in the course of an interval Dr. R. Ashleigh Clegg spoke of ambulance work in Lincoln. He appealed to the women in the audience to offer themselves for training, and undertook that they should be trained free of charge in London for the work of village nursing. He also appealed for the names of volunteers desiring to offer themselves for first-aid, or to help the nurses in an emergency. We hope to hear this interesting demonstration has been repeated in other places.

The prospect of a coal strike is causing much anxiety in institutions unprovided with large storage. With coal at its present famine price it did not need that to make the winter outlook as regards heating distinctly grave. To reduce the amount of coal used in every establishment to a minimum there is no better way than to introduce wood and coke fires into the wards and sitting-rooms. In London the price of logs is prohibitive. But in many parts of England wood can be obtained at a fairly easy rate, to be chopped on the premises, and landowners would readily respond to requests for crooked branches and the trimmings of timber to keep the hospital fires burning. It is true that coke is as dear as coal, but only a small quantity is required with the logs to build up magnificent fires. Though rather more troublesome, it has been clearly demonstrated that the wood and coke fire is a distinct economy.

It is perennially interesting to know what becomes of nurses after they have received the certificates of their training-schools, for it is probable that the wastage to the profession in respect of qualified nurses who do not continue to nurse is more serious than in other occupations. The analysis given in some hospital reports of the nurses leaving during the year throws considerable light on the subject. In the last report of the Edinburgh Royal Infirmary, for instance, particulars are given of 102 members of the nursing staff who left during the year. Twenty-one of these were probationers, of whom sixteen were on trial and not found suitable. Of the eighty-one certificated nurses five retired and were pensioned after periods of service varying from twenty-two to thirty-five years; twenty took up private nursing; nineteen went back to their homes;

**ROUND THE HOSPITALS—(*continued*).**

twelve took posts in other hospitals; eleven went on to take maternity training; six left to be married; four joined the Q.A.I.M.N.S.; one became a medical student; one a health visitor; one joined Q.V.J.I.; and one went abroad. The average of nurses on the staff is 306. Thus there was a loss to the profession of about 40 per cent. of those who left in the year, and of about 10 per cent. of the entire staff.

---

The matron's post at the Guest Hospital, Dudley, does not appear to be altogether a bed of roses, judging from the fact that it is again vacant after a brief occupancy of two years. Entering on their work with high hopes, successive matrons appear to find insuperable difficulties in the way of modernising conditions, and as this is the third resignation within three years the managers would perhaps be wise to look a little more closely into the causes which have produced this sense of discouragement.

---

A remarkably good appeal, well printed and put together, is in circulation on behalf of the Edith Cavell Homes, reproducing a beautiful likeness of Edith Cavell from the painting in possession of Queen Alexandra, and we note that copies of this likeness can be had at a very moderate price. The Homes now open for the reception of nurses requiring rest are: Coombe Head, Haslemere (freehold); Little Wych, Bridport; Raven House, Adderley; the Mythe Grange, Tewkesbury; Winton House, Richmond; The Hollies, West Norwood (freehold); The Cross Ways, Windermere (freehold). Arrangements have also been made for nurses to be received temporarily in other places, and thus about 600 nurses can be provided with a month's rest every year. The Council find, however, that this accommodation is inadequate. To meet the requirements of the nursing service there should be room for at least 500 more nurses yearly. On October 12 an effort is to be made to raise a sum sufficient permanently to endow the three freehold homes named above, for which about £30,000 would be required. Nurses who will co-operate by assisting to sell violets on the Memorial Day in the London area will be welcomed, and should communicate with Mrs. Adair, Edith Cavell Homes for Nurses, 25 Victoria Street, Westminster. White card-board trays have been prepared for the flowers which will be sold singly or in bunches.

---

The "Not Forgotten" Association is rapidly helping to remove the stigma of ingratitude towards the wounded from Londoners, but it needs further and continuous help to ensure the carrying into effect of its excellent aims. Lady Haig, Lady Beatty, and Lady Trenchard, the presidents, have forwarded £100 to Miss Marta Cunningham, the honorary organising secretary, to form a starting fund towards the current expenses. Fruit, chocolate, cigarettes, and tobacco, are supplied to the wounded, and entertainments are organised for those able to enjoy them outside. Parties were given lately by three hostesses to the wounded from Denmark Hill, Millbank, and Du Cane Road Hospitals, and this week Sir Henry and Lady Samuelson entertain thirty-six Roehampton men at Cobham, while Lady Goschen is giving an entertainment at the headquarters of the Association, Miss Marta Cunningham's Studio, 86 Ladbrooke Road, W. 11. The County Director B.R.C.S. and Order of St. John has undertaken the conveyance of all the men. There should be hundreds competing for the honour of receiving these men in their homes, and it may be that in small groups, regarded as individuals, they would enjoy themselves even more than in large parties.

---

Energetic steps are being taken in Australia to look after the men on whom war has left its traces, though it may not have brought them total disablement. In the most sparsely populated regions where returning soldiers are settling on land granted by the State the nurses follow on, and, through the medium of the Bush Nursing Associations, now undergoing great expansion, are available for their needs no less than for those of their families. The Anzac Hotel in South Australia promises to be a great success. A fine property with good mansion and extensive grounds has been provided at Glenelg, and the nursing staff is made up of Miss Donnell as matron, six sisters and staff nurses, and two V.A.D.s. There is every facility for interesting the patients in outdoor pursuits, and their comfort is well attended to.

---

At Bath Health Committee the superintendent of the maternity home called attention to the difficulties experienced in obtaining suitable applicants for the position of midwives and nurses, and suggested that the salary scale should be revised. The Committee accordingly proposed the following new scale:—*Midwives*: £150 per annum on appointment, rising by annual increments of £10 to a maximum of £180 per annum, with uniform. *Health Visitors*: £140 on appointment, rising by annual increments of £10 to a maximum of £170 per annum, with an allowance of £10 per annum for uniform.

---

Durham County Council has agreed to a report of the County Medical Officer and County Superintendent Health Visitor on study leave for health visitors. The scheme is that health visitors who have proved themselves anxious to learn and are likely to make first-class officers shall be paid one-half salary for study leave for training courses at approved institutions. A health visitor who takes three months' leave will be required to remain in the service of the County Council one complete year afterwards, and those taking six months' leave will be required to remain two complete years, an agreement being signed and a penalty imposed for non-compliance.

